# Supplementary material for: Phosphorylation of plasma membrane H+-ATPase Thr881 participates in light-induced stomatal opening
Source: Nat Commun. 2024 Feb 20;15:1194. doi: 10.1038/s41467-024-45248-5 (PMC10879185; doi:10.1038/s41467-024-45248-5)
Supplement: Supplementary file 1 — Supplementary information [file 41467_2024_45248_MOESM1_ESM.pdf]

Supplementary information for

Phosphorylation of plasma membrane H^+-ATPase Thr881 participates in light-induced stomatal opening

Yuki Hayashi[1†], Kohei Fukatsu[1†], Koji Takahashi[1,2†], Satoru N. Kinoshita[1], Kyohei Kato[1], Taku Sakakibara[1], Keiko Kuwata[2], Toshinori Kinoshita[1,2]*

[1]Graduate School of Science, Nagoya University, Chikusa, Nagoya, Japan

[2]Institute of Transformative Bio-Molecules (WPI-ITbM), Nagoya University, Chikusa, Nagoya, Japan

[†]These authors contributed equally.

*Toshinori Kinoshita Email: kinoshita@bio.nagoya-u.ac.jp

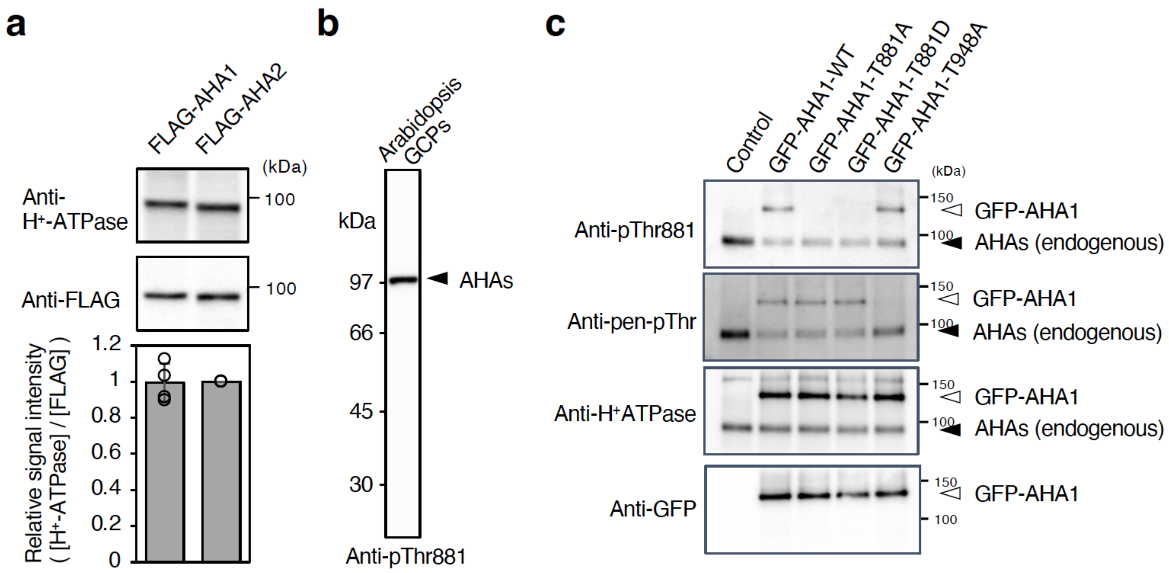

Supplementary Fig. 1 Specificity of anti-H$^+$-ATPase, anti-pThr881 and anti-pen-pThr antibodies for detection of PM H$^+$-ATPases. a, Detection of FLAG-AHA1 and FLAG-AHA2 by anti-H$^+$-ATPase and anti-FLAG antibodies. FLAG-AHA1 and FLAG-AHA2 synthesized by *in vitro* translation system with wheat germ extract (NU Protein) were detected by the immunoblot analysis using anti-H$^+$-ATPase and anti-FLAG. Lower graph shows the relative signal intensity which is calculated by dividing the signal intensity of the bands detected with anti-H$^+$-ATPase by that of the band detected with anti-FLAG antibody. Data are mean $\pm$ SD of four independent experiments. **b**, Detection of phosphorylated Thr881 of PM H$^+$-ATPase in Arabidopsis GCPs by anti-pThr881 antibody. Proteins extracted from *Arabidopsis* GCPs were subjected to SDS-PAGE and immunoblot analysis using anti-pThr881 antibody produced in this study. Arrowhead shows the position of AHAs (Arabidopsis PM H$^+$-ATPases). Numbers at left indicate molecular weight markers. **c**, Detection of GFP-AHA1-WT, -T881A, -T881D and -T948A with anti-pThr881 and anti-pen-pThr antibodies. GFP-AHA1-WT, -T881A, -T881D and -T948A transiently expressed in mesophyll cell protoplasts were detected by the immunoblot analysis using anti-pThr881, anti-pen-pThr, anti-H$^+$-ATPase and anti-GFP antibodies. White and black arrowheads show the positions of GFP-AHA1 and endogenous AHAs (Arabidopsis H$^+$-ATPases), respectively.

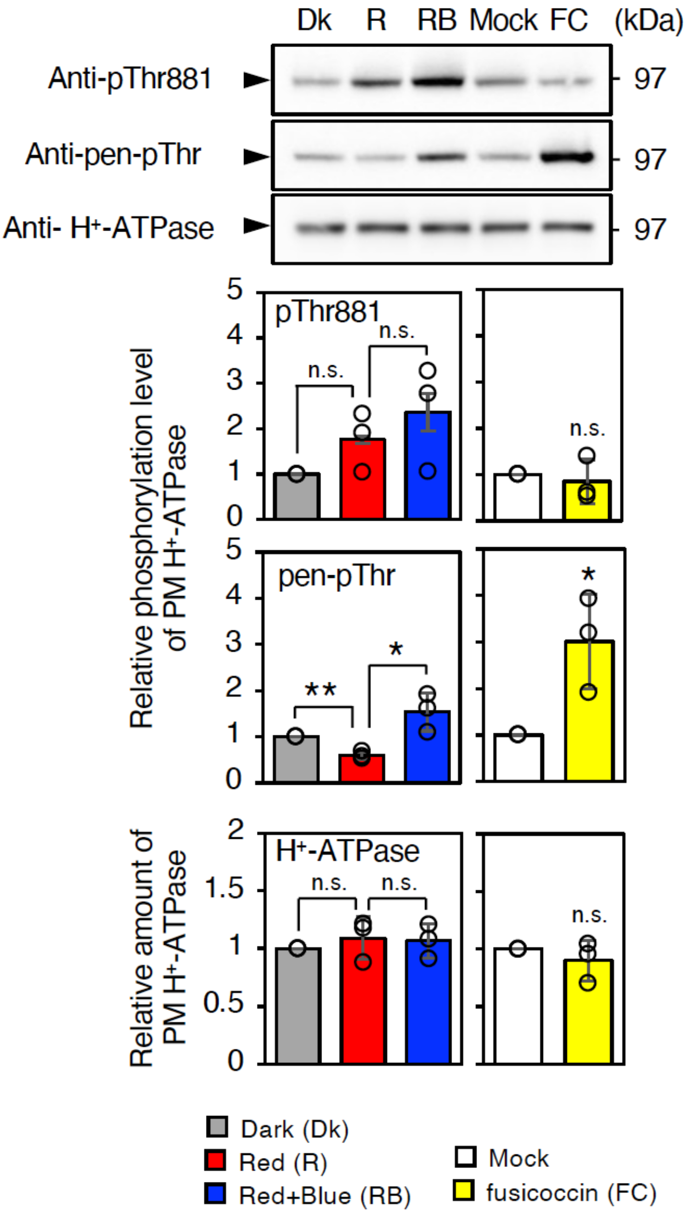

Supplementary Fig. 2 Phosphorylation of PM H$^+$-ATPase in GCPs from *Arabidopsis thaliana*. a, GCPs were isolated from *Arabidopsis thaliana* Col-0 and kept in the dark for 1 hr. Dark-adapted GCPs were illuminated with red light and blue light or treated with FC as described in Table 1 and Supplementary Table 3. Data are mean ± SD of three independent experiments. The asterisk indicates statistically significant difference (one-tailed Student's *t* test: *, $P < 0.05$; **, $P < 0.001$; n.s., Not significant, $P > 0.05$). Others are same as in Fig. 2.

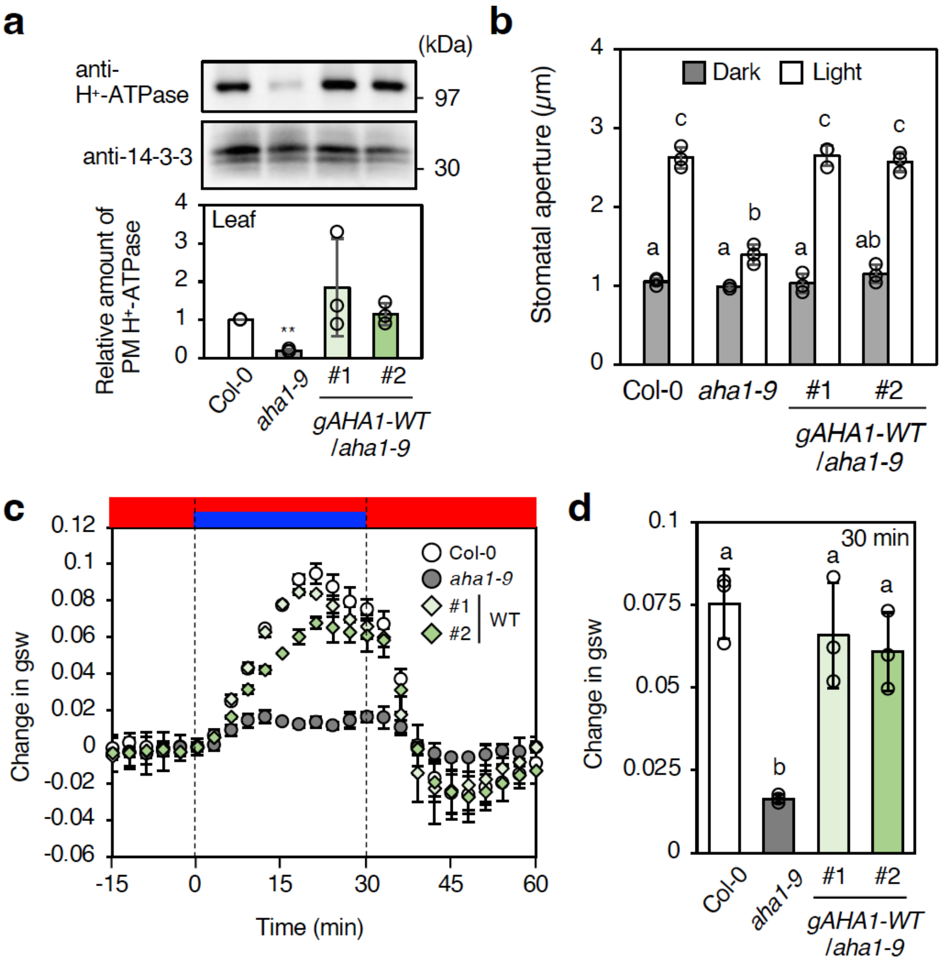

Supplementary Fig. 3 Phenotypes of AHA1 complementation plants. a, Expression of wild-type AHA1 in *aha1-9* mutant (*gAHA1-WT/aha1-9*). Others are same as in **Fig. 3a**. **b**, Light-induced stomatal opening in g*AHA1-WT* plants. Data are mean ± SD (n=3, independent experiments); measurement of 30 stomata in each experiment. Different letters indicate statistically significant differences among means (ANOVA with Tukey's test: $P < 0.05$). Other details are the same as in **Fig. 3c**. **c**, Light-dependent changes in stomatal conductance. Leaves from dark-acclimated plants were illuminated with red light (Red: 600 μmol m^{-2} s^{-1}) and/or blue light (60 μmol m^{-2} s^{-1}) as indicated. The gsw values were calculated as the change from the value at the start of blue light irradiation. Data are mean ± SD of three independent experiments. **d**, Stomatal conductance at 30 min after the start of blue light illumination. Data were obtained from **c**. Different letters indicate statistically significant differences among means (ANOVA with Tukey's test: $P < 0.01$).

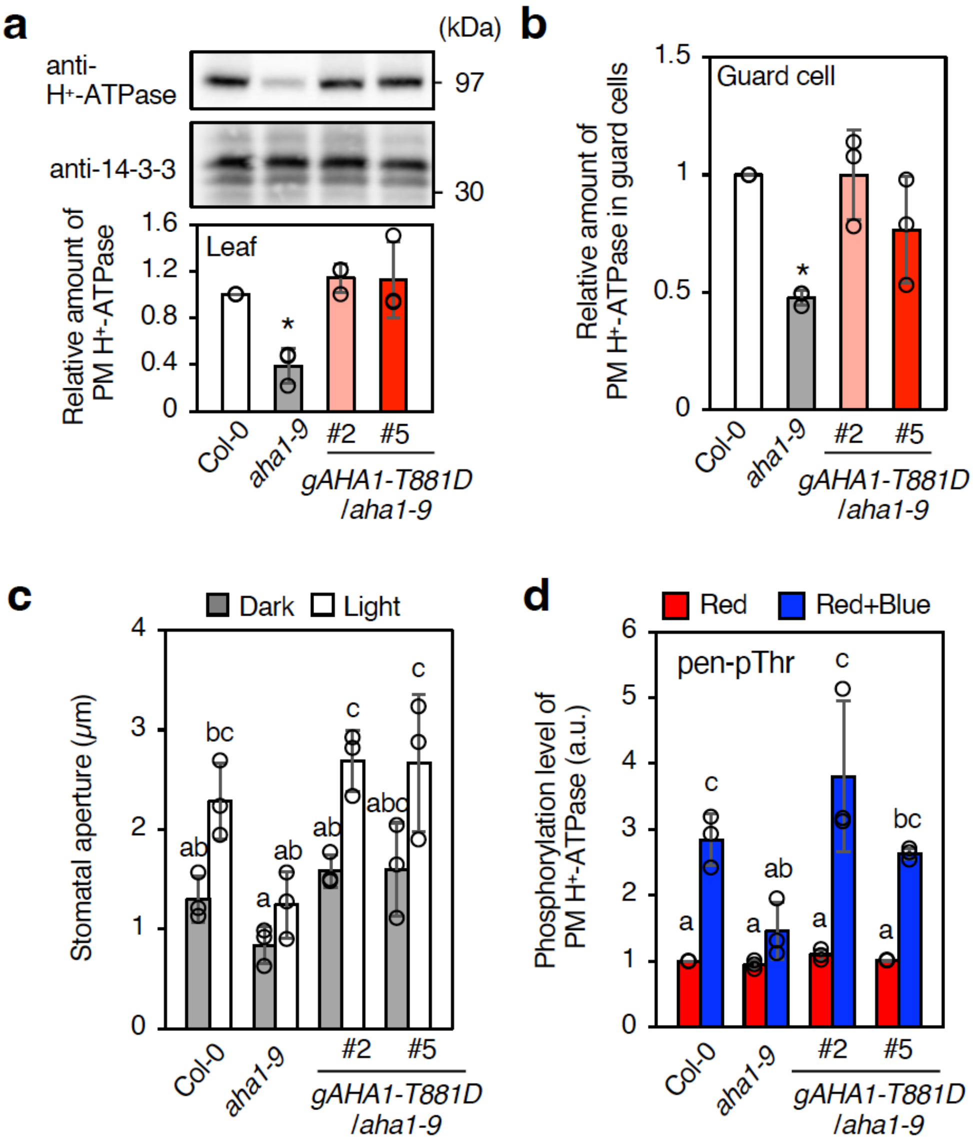

Supplementary Fig. 4 Effect of phospho-mimic mutation of Thr881 in PM H^+-ATPase on stomatal responses. a, Expression of Thr881 phospho-mimic form (T881D) of AHA1 in *aha1-9* mutant (*gAHA1-T881D/aha1-9*). Data are mean ± SD of three independent experiments. Asterisk indicates a significant statistical difference relative to Col-0 (two-tailed Student's *t* test: *, $P < 0.05$). Other details are the same as in **Fig. 3a**. **b**, Amount of PM H^+-ATPase in guard cells of *gAHA1-T881D* plants. Data are mean ± SD of three independent experiments. Asterisk indicates a significant statistical difference relative to Col-0 (two-tailed Student's *t* test: *, $P < 0.0001$). Others are same as in **Fig. 3b**. **c**, Light-induced stomatal opening in g*AHA1-T881D* plants. Data are mean ± SD (n=3, independent experiments); measurement of

30 stomata in each experiment. Different letters indicate statistically significant differences among means (ANOVA with Tukey's test: $P < 0.05$). Others are same as in **Fig. 3c**. **d**, Immunohistochemical detection of the pen-Thr phosphorylation in guard cells in response to blue light in *gAHA1-T881D* plants. Data are mean ± SD of three independent experiments. Different letters indicate statistically significant differences among means (ANOVA with Tukey's test: $P < 0.05$). Other details are the same as in Fig. 1a.

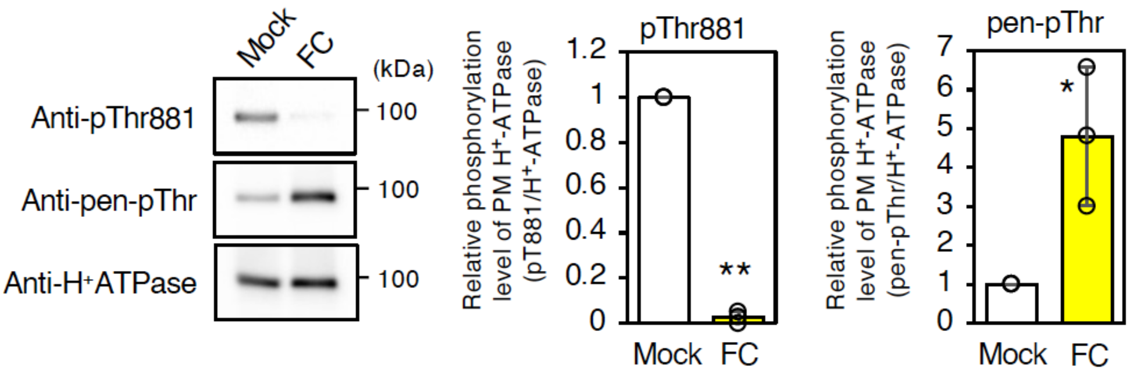

Supplementary Fig. 5 Phosphorylation of PM H^+-ATPases in response to fusicoccin in the etiolated hypocotyl segments. Hypocotyl segments from 3-day-old Arabidopsis etiolated seedlings were treated with DMSO (Mock) or 10 µM FC (FC) in the dark for 30 min. Data are mean ± SD of three independent experiments. The asterisk indicates statistically significant difference (two-tailed Student's *t* test: *, $P < 0.05$; **, $P < 0.0001$). Others are same as in **Fig. 4.**

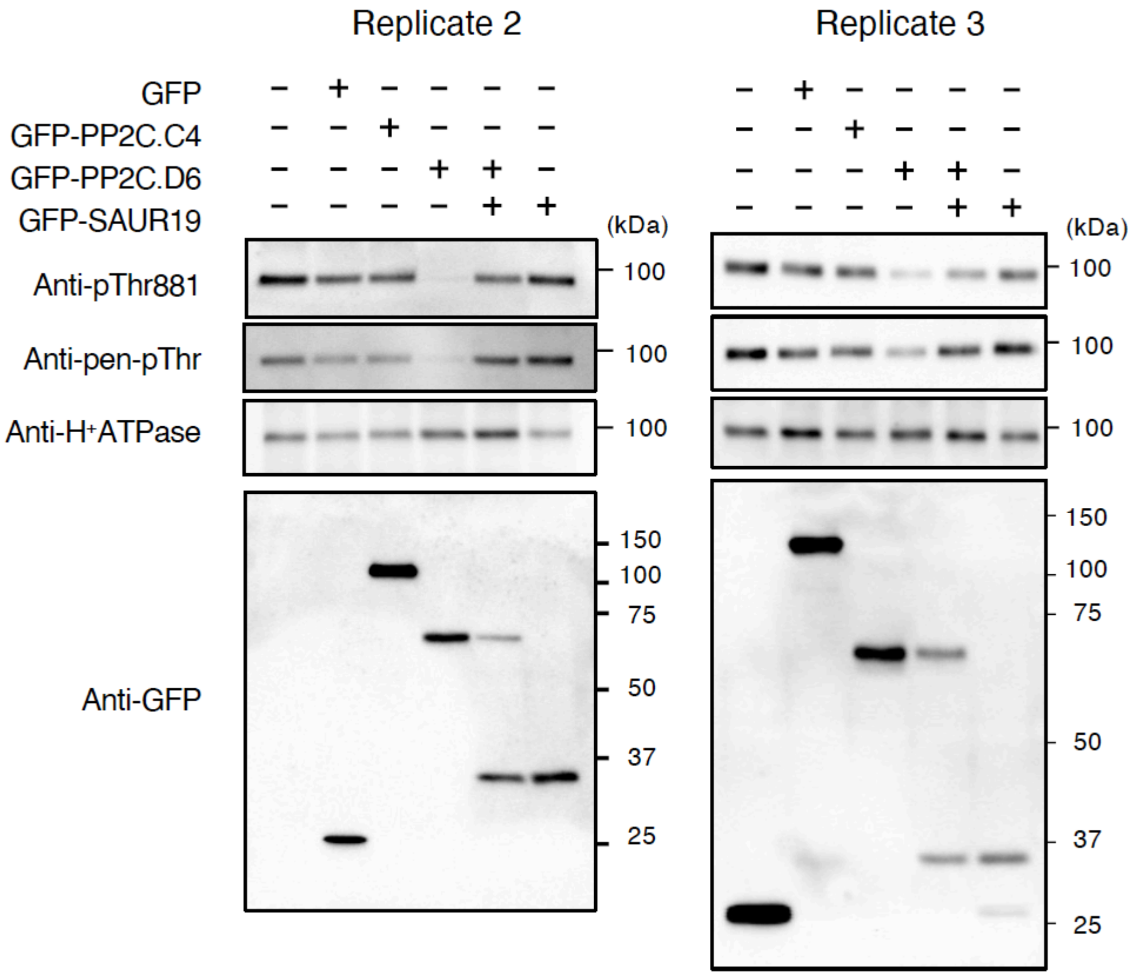

Supplementary Fig. 6 Transient expression of *GFP-PP2Cs* and *GFP-SAUR19* in the mesophyll cell protoplasts. The results of Replicate 2 and 3 as in Fig. 5b are shown.

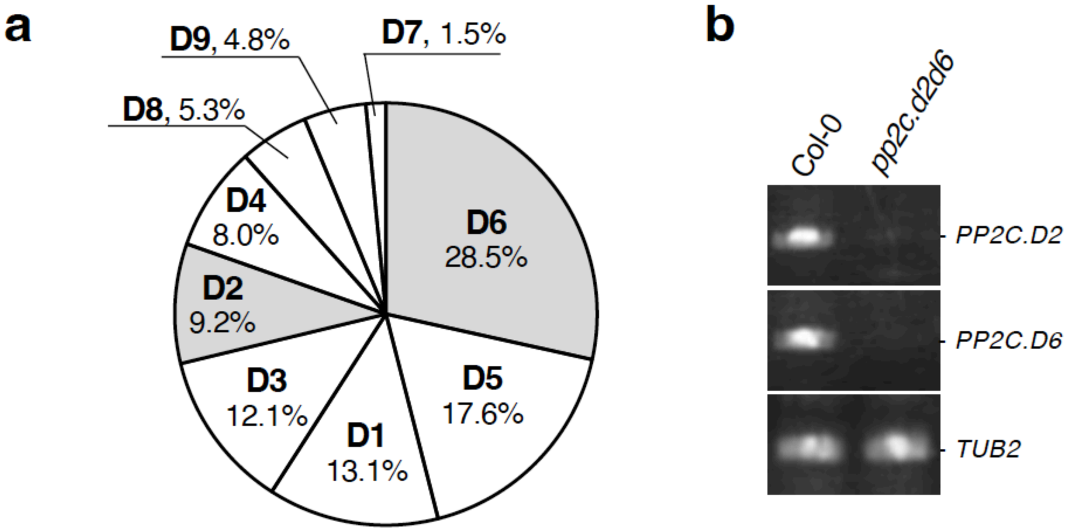

Supplementary Fig. 7 Expression levels of PP2C.Ds. a, Relative expression levels of PP2C.Ds (D1; At5g02760, D2; At3g17090, D3; At3g12620, D4; At3g55050, D5; At4g38520, D6; At3g51370, D7; At5g66080, D8; At4g33920, D9; At5g06750) in Arabidopsis Col-0 leaves. Data were obtained from Arabidopsis eFP browser (http://bar.utoronto.ca/efp/cgi-bin/efpWeb.cgi). **b**, Expression of *PP2C.D2* and *PP2C.D6* analyzed by RT-PCR in Col-0 and *pp2c.d2d6*. Total RNA was extracted from rosette leaves of 4-week-old plants. *TUB2* was used as an internal control. The primers are listed in Supplementary Table 6.

Supplementary Table 1 Phosphoproteome analysis of *Vicia* GCPs illuminated with red and blue light. PSMs of each phospho-peptides from vfphot1a, vfphot1b, BLUS1, LMBR1-like membrane protein and ARM repeat superfamily protein are indicated. The numbers in Phospho-site of vfphot1a, vfphot1b correspond to the amino acid number of Arabidopsis phot1. The number in Phospho-site of BLUS1 corresponds to the amino acid number of Arabidopsis BLUS1. Experiments repeated on three occasions are shown.

Contig	Phospho-site	PSMs								
		Rep.1			Rep.2			Rep.3		
		Dk	R	R+B	Dk	R	R+B	Dk	R	R+B
vfphot1a	Ser-350	0	0	9	0	0	5	5	5	13
vfphot1b	Ser-350	0	0	15	0	0	10	0	0	11
BLUS1	Ser-348	8	19	65	11	13	47	13	13	67
LMBR1-like membrane protein	Ser-720	0	0	5	0	0	4	0	0	5
ARM repeat superfamily protein	Ser-7	0	0	12	2	2	11	0	0	10

Supplementary Table 2 Phosphoproteome analysis of *Vicia* GCPs illuminated with red and blue light. The results of Replicate 2 and 3 as in Table 1 are shown.

Replicate 2

Phospho-site	Contig	PSMs			Fold change	
		Dk	R	R+B	([R]/[Dk])	([R+B]/[R])
Thr881	VHA1	10	98	136	9.80	1.39
	Unnamed isoform	0	24	35	-	1.46
Ser899	VHA1	11	16	18	1.45	1.13
	Unnamed isoform	7	6	3	0.86	0.50
Thr942	VHA1/Unnamed isoform	5	16	23	3.20	1.44
Thr948 (pen-Thr)	VHA1/Unnamed isoform	31	99	136	3.19	1.37

Replicate 3

Phospho-site	Contig	PSMs			Fold change	
		Dk	R	R+B	([R]/[Dk])	([R+B]/[R])
Thr881	VHA1	11	22	70	2.00	3.18
	Unnamed isoform	1	0	16	0	-
Ser899	VHA1	14	19	19	1.36	1.00
	Unnamed isoform	17	16	11	0.94	0.69
Thr942	VHA1/Unnamed isoform	10	9	22	0.90	2.44
Thr948 (pen-Thr)	VHA1/Unnamed isoform	44	51	113	1.16	2.22

Supplementary Table 3 Phosphoproteome analysis of *Vicia* GCPs treated with Fusicoccin (FC). Dark-adapted GCPs were treated with DMSO (Mock) or 10 µM FC (FC) for 5 min in the dark. PSMs of each phospho-peptides from VHA1 and unnamed isoform and fold change are indicated (-; infinity) are indicated. The numbers in Phospho-site correspond to the amino acid number of AHA1. Experiments repeated on three occasions are shown.

Replicate 1

Phospho-site	Contig	PSMs		Fold change
		DMSO	FC	([FC]/[DMSO])
Thr881	VHA1	11	32	2.91
	Unnamed isoform	0	4	-
Ser899	VHA1	45	55	1.22
	Unnamed isoform	0	3	-
Thr942	VHA1/Unnamed isoform	19	20	1.05
Thr948 (pen-Thr)	VHA1/Unnamed isoform	80	161	2.01

Replicate 2

Phospho-site	Contig	PSMs		Fold change
		DMSO	FC	([FC]/[DMSO])
Thr881	VHA1	1	12	12.00
	Unnamed isoform	0	0	-
Ser899	VHA1	17	14	0.82
	Unnamed isoform	0	0	-
Thr942	VHA1/Unnamed isoform	0	2	-
Thr948 (pen-Thr)	VHA1/Unnamed isoform	29	72	2.48

Replicate 3

Phospho-site	Contig	PSMs		Fold change
		DMSO	FC	([FC]/[DMSO])
Thr881	VHA1	0	5	-
	Unnamed isoform	0	0	-
Ser899	VHA1	33	38	1.15
	Unnamed isoform	5	12	2.40
Thr942	VHA1/Unnamed isoform	4	3	0.75
Thr948 (pen-Thr)	VHA1/Unnamed isoform	10	15	1.50

Supplementary Table 4 List of primers for construction of *pCAMBIA1300/gAHA1*.

Primer Name	Sequence (5' to 3')
for cloning of genomic DNA fragment of AHA1	
gAHA1-Fw1	GGCCAGTGCCAAGCTctactacacatacatgagtcttaaatc
gAHA1-Rv1	CCGGGGATCCTCTAGCCATATCTTTGGACGTGACTCG
for PCR-based site-directed mutagenesis	
AHA1-T881-Fw	TTGCACGGTCTGCAGCCAAAAGAAGATG
AHA1-T881A-Rv	CTGCAGACCGTGCAATGcCCTTTGAG
AHA1-T881D-Rv	CTGCAGACCGTGCAAgtcCCTTTGAG
AHA1-T948-Fw	GTGTAGTTGGAGTTGCACAACAACACAAAC
AHA1-T948A-Rv	CAACTCCAACTACACAGcGTAGTGATGTCC
gAHA1-Fw2	attcctttttagGTGGTACCCGAG
gAHA1-Rv2	ACGAATTCGAGCTCGGTACC

Supplementary Table 5 List of primers for construction of *pUC18/p35S:mGFP::Linker::AHA1:nos3'*.

Primer Name	Sequence (5' to 3')
for pUC18/p35S:mGFP::Linker::AHA1:nos3'	
Linker-Fw	TCTGGTGGTGGAGGATCGCTCGAGTAATGTACAAGTAAAGCGGCCGC
mGFP-Rv1	CACGCCGTAGGTGAAGGTG
mGFP-Fw1	TTCACCTACGGCGTGatgTGCTTCAGC
mGFP-Rv2	GGACTGGGTGCTCAGGTAG
mGFP-Fw2	CTGAGCACCCAGTCCaaaCTGAGCAAAGAC
Linker-Rv	TCCTCCACCACCAGATCCACCTCCACCCTTGTACAGCTCGTCCATGCC
Linker-AHA1-Fw	TGGAGGATCGctCgAaATGTCAGGTCTCGAAGATATCAAG
AHA1-XhoI-Rv1	GCTCTcgagGTGAGAACGGCACTGATGATG
AHA1-XhoI-Fw1	TCTCACctcgAGAGCTATCTTCCAGAGAAT
AHA1-XhoI-Rv2	TGTACATTActcgagCTACACAGTGTAGTGATGTCCTG
for PCR-based site-directed mutagenesis	
AHA1-XhoI-Fw2	CCGTTCTCACctcgAGAGC
AHA1-T881A-Rv	CTGCAGACCGTGCAATGcCCTTTGAG
AHA1-T881-Fw	TTGCACGGTCTGCAGCCAAAAGAAGATG
AHA1-WT-Rv	TTGTACATTACTCGAGCTACACAGTGTAGTGATGTCCTGC
AHA1-T881D-Rv	CTGCAGACCGTGCAAgtcCCTTTGAG
AHA1-T948A-Rv2	TTGTACATTACTCGAGCTACACAGCGTAGTGATGTCCTGC

Supplementary Table 6 List of primers for RT-PCR.

Gene	Fw/Rv	Sequence (5' to 3')
PP2C.D2	Fw	TTTTGGGGGTTAGGGTTTTC
	Rv	AGTTGATGTGGCCTCTCGAT
PP2C.D6	Fw	TGCTTTGATGTGTATCAGTTGAC
	Rv	CCGTTATCGTTGGTTCCCCA
TUB2	Fw	CATTGTTGATCTCTAAGATCCGTG
	Rv	TACTGCTGAGAACCTCTTGAG
