# Supplementary material for: Phosphorylation of plasma membrane H+-ATPase Thr881 participates in light-induced stomatal opening
Source: Nat Commun. 2024 Feb 20;15:1194. doi: 10.1038/s41467-024-45248-5 (PMC10879185; doi:10.1038/s41467-024-45248-5)
Supplement: Supplementary file 3 — Reporting Summary [file 41467_2024_45248_MOESM3_ESM.pdf]

nature portfolio

Corresponding author(s): Toshinori Kinoshita

Last updated by author(s): Dec 29, 2023

Reporting Summary

Nature Portfolio wishes to improve the reproducibility of the work that we publish. This form provides structure for consistency and transparency in reporting. For further information on Nature Portfolio policies, see our Editorial Policies and the Editorial Policy Checklist.

Statistics

For all statistical analyses, confirm that the following items are present in the figure legend, table legend, main text, or Methods section.

n/a	Confirmed	
☐	☒	The exact sample size (*n*) for each experimental group/condition, given as a discrete number and unit of measurement
☐	☒	A statement on whether measurements were taken from distinct samples or whether the same sample was measured repeatedly
☐	☒	The statistical test(s) used AND whether they are one- or two-sided<br>*Only common tests should be described solely by name; describe more complex techniques in the Methods section.*
☒	☐	A description of all covariates tested
☒	☐	A description of any assumptions or corrections, such as tests of normality and adjustment for multiple comparisons
☐	☒	A full description of the statistical parameters including central tendency (e.g. means) or other basic estimates (e.g. regression coefficient) AND variation (e.g. standard deviation) or associated estimates of uncertainty (e.g. confidence intervals)
☒	☐	For null hypothesis testing, the test statistic (e.g. F, t, r) with confidence intervals, effect sizes, degrees of freedom and P value noted<br>*Give P values as exact values whenever suitable.*
☒	☐	For Bayesian analysis, information on the choice of priors and Markov chain Monte Carlo settings
☒	☐	For hierarchical and complex designs, identification of the appropriate level for tests and full reporting of outcomes
☒	☐	Estimates of effect sizes (e.g. Cohen's *d*, Pearson's *r*), indicating how they were calculated

Our web collection on statistics for biologists contains articles on many of the points above.

Software and code

Policy information about availability of computer code

Data collection	Microsoft Excel for Mac was used to collect the data.
Data analysis	Microsoft Excel for Mac was used for statistical analyses and drawing graphs.

For manuscripts utilizing custom algorithms or software that are central to the research but not yet described in published literature, software must be made available to editors and reviewers. We strongly encourage code deposition in a community repository (e.g. GitHub). See the Nature Portfolio guidelines for submitting code & software for further information.

Data

Policy information about availability of data

All manuscripts must include a data availability statement. This statement should provide the following information, where applicable:

- Accession codes, unique identifiers, or web links for publicly available datasets
- A description of any restrictions on data availability
- For clinical datasets or third party data, please ensure that the statement adheres to our policy

All data supporting the findings of this study are available within the main text and its Supplementary Information files. Raw data that supports the findings are available in the Source File or from the corresponding author upon reasonable request.

Research involving human participants, their data, or biological material

Policy information about studies with human participants or human data. See also policy information about sex, gender (identity/presentation), and sexual orientation and race, ethnicity and racism.

Reporting on sex and gender	*Use the terms sex (biological attribute) and gender (shaped by social and cultural circumstances) carefully in order to avoid confusing both terms. Indicate if findings apply to only one sex or gender; describe whether sex and gender were considered in study design; whether sex and/or gender was determined based on self-reporting or assigned and methods used. Provide in the source data disaggregated sex and gender data, where this information has been collected, and if consent has been obtained for sharing of individual-level data; provide overall numbers in this Reporting Summary. Please state if this information has not been collected. Report sex- and gender-based analyses where performed, justify reasons for lack of sex- and gender-based analysis.*
Reporting on race, ethnicity, or other socially relevant groupings	*Please specify the socially constructed or socially relevant categorization variable(s) used in your manuscript and explain why they were used. Please note that such variables should not be used as proxies for other socially constructed/relevant variables (for example, race or ethnicity should not be used as a proxy for socioeconomic status). Provide clear definitions of the relevant terms used, how they were provided (by the participants/respondents, the researchers, or third parties), and the method(s) used to classify people into the different categories (e.g. self-report, census or administrative data, social media data, etc.) Please provide details about how you controlled for confounding variables in your analyses.*
Population characteristics	*Describe the covariate-relevant population characteristics of the human research participants (e.g. age, genotypic information, past and current diagnosis and treatment categories). If you filled out the behavioural & social sciences study design questions and have nothing to add here, write "See above."*
Recruitment	*Describe how participants were recruited. Outline any potential self-selection bias or other biases that may be present and how these are likely to impact results.*
Ethics oversight	*Identify the organization(s) that approved the study protocol.*

Note that full information on the approval of the study protocol must also be provided in the manuscript.

Field-specific reporting

Please select the one below that is the best fit for your research. If you are not sure, read the appropriate sections before making your selection.

☒ Life sciences ☐ Behavioural & social sciences ☐ Ecological, evolutionary & environmental sciences

For a reference copy of the document with all sections, see nature.com/documents/nr-reporting-summary-flat.pdf

Life sciences study design

All studies must disclose on these points even when the disclosure is negative.

Sample size	No statistical methods were used to predetermine sample size. Sample sizes were chosen to produce biologically meaningful data for comparison purposes, based on previous experiments of out laboratory and others using the physiological and biochemical methods described.
Data exclusions	No data were excluded from the analyses.
Replication	All experiments were conducted with at least two independent biological replicates and gave similar results.
Randomization	Sample randomization was not relevant for physiological and biochemical analyses in this study.
Blinding	Blinding was not performed. Group allocation according to genotype was done before data collection. Blinding was not possible because the same investigator processed the samples and analyzed the data.

Reporting for specific materials, systems and methods

We require information from authors about some types of materials, experimental systems and methods used in many studies. Here, indicate whether each material, system or method listed is relevant to your study. If you are not sure if a list item applies to your research, read the appropriate section before selecting a response.

Materials & experimental systems

n/a	Involved in the study
	☒ Antibodies
☒	Eukaryotic cell lines
☒	Palaeontology and archaeology
☒	Animals and other organisms
☒	Clinical data
☒	Dual use research of concern
	☒ Plants

Methods

n/a	Involved in the study
☒	ChIP-seq
☒	Flow cytometry
☒	MRI-based neuroimaging

Antibodies

Antibodies used

Rabbit anti-H+-ATPase polyclonal antibody (1:3000 dilution), rabbit anti-pen-pThr polyclonal antibody (1:3000 dilution), rabbit anti-pThr881 polyclonal antibody (1:3000 dilution), rabbit anti-14-3-3-polyclonal antibody (1:3000 dilution), and rabbit anti-BHP polyclonal antibody (1:3000 dilution) have been produced by our research groups. Mouse anti-GFP-monoclonal antibody (1:3000 dilution) and mouse anti-FLAG monoclonal antibody (1:3000 dilution) were purchased from Roche and Sigma-Aldrich, respectively. Goat anti-rabbit IgG antibody-Alexa Fluor 488 (1:500 dilution, #A-11034) was purchase from Thermo Fisher Scientific, Inc. Goat anti-rabbit IgG antibody conjugated with HRP (1:3000 dilution, #1706515) and goat anti-mouse IgG antibody conjugated with HRP (1:3000 dilution, #1706516) were purchased from Bio-Rad.

Validation

The antibody profiles and validations are available in the following references.

<Rabbit anti-H+-ATPase polyclonal antibody>
Polyclonal antibody against the conserved catalytic domain of the plasma membrane H+-ATPase of Arabidopsis (AHA2) were raised in rabbit. (Hayashi Y, Nakamura S, Takemiya A, Takahashi Y, Shimazaki K and Kinoshita T. Plant Cell Physiol. 51: 1186-1196, 2010)

<Rabbit anti-pen-pThr polyclonal antibody>
Polyclonal antibody against the penultimate phospharylated Thr947 of the plasma membrane H+-ATPase of Arabidopsis (AHA2) were raised in rabbits using the phosphorylated synthetic peptide (CIETPSHYpTV) as an antigen. (Hayashi Y, Nakamura S, Takemiya A, Takahashi Y, Shimazaki K and Kinoshita T. Plant Cell Physiol. 51: 1186-1196, 2010)

<Rabbit anti-pThr881 polyclonal antibody>
This antibody was produced in this study.

<Rabbit anti-14-3-3 polyclonal antibody>
Kinoshita T and Shimazaki K. Blue light activates the plasma membrane H+-ATPase by phosphorylation of the C-terminus in stomatal guard cells. EMBO J. 18: 5548-5558 (1999)

<Rabbit anti-BHP polyclonal antibody>
Hayashi M, Inoue S, Ueno Y and Kinoshita T. A Raf-like protein kinase BHP mediates blue light-dependent stomatal opening. Sci. Rep. 7: 45586 (2017)

<Mouse anti-GFP monoclonal antibody>
Anti-Green Fluorescent Protein antibody from mouse IgG1K (clone 7.1 and 13.1), Roche Lot#10126200
https://www.sigmaaldrich.com/JP/en/product/roche/11814460001

<Mouse anti-FLAG monoclonal antibody>
Anti-FLAG (DYKDDDK) antibody from clone M2, purified immunogloblin (Purified IgG1 subclass), Sigma-Aldrich #F3165
https://www.sigmaaldrich.com/JP/en/product/sigma/f3165

Plants

Seed stocks

Vicia faba (broad bean; Ryosai Issun) seeds were purchased from Nakahara Saisyujyo, Japan. Arabidopsis Columbia-0 (Col-0) plants were used as the wild-type. Col-0 is a background ecotype of T-DNA insertion mutants of aha1–9 (SAIL_1285_D12; At2G18960), psy1r-1 (SALK_072802; At1g72300), pskr1-2 (SAIL_673_H07; At2g02220), pskr2 (SALK_024464; At5g53890), pp2c.d2 (SALK_203806; At3g17090) and pp2c.d6 (SAIL_171H03; At3g51370). The T-DNA insertion mutants were obtained from the Arabidopsis Biological Resource Center (Ohio State University). gl1, Columbia ecotype, is a background plant of phot1-5 phot2-1. Two independent T3 homozygous lines were used for each experiment.

Novel plant genotypes

A genomic DNA fragment of AHA1 containing the promoter and terminator regions (4,188 bp upstream from the start codon to 1,106 bp downstream from the stop codon) was cloned into the Hind III–Xba I site of pCAMBIA1300. This resultant plasmid was designated pCAMBIA1300/gAHA1-WT. pCAMBIA1300/gAHA1-T881A, pCAMBIA1300/gAHA1-T881D, and pCAMBIA1300/gAHA1-T948A were constructed by PCR-based site-directed mutagenesis using pCAMBIA1300/gAHA1-WT as the PCR templates. All constructed vectors were transformed into the aha1–9 mutant by floral dipping using Agrobacterium tumefaciens GV3101.

Authentication

Homozygous lines of T-DNA insertion mutants and transgenic plants were isolated based on kanamycin screening and PCR genotyping. To avoid effects of T-DNA insertion (position-effects) we used at least two independent lines regarding the transgenic plants novelly generated in this study.
